# The Effect of Organizational Support Climate on Employees’ Positive Deviance: A Parallel Mediation Model

**DOI:** 10.3390/bs16010053

**Published:** 2025-12-28

**Authors:** Yuqing Meng, Mingpeng Huang

**Affiliations:** 1 Seoul School of Integrated Sciences and Technologies, Seoul 03767, Republic of Korea; 2 Business School, University of International Business and Economics, Beijing 100029, China; hmp@uibe.edu.cn

**Keywords:** positive deviance, organizational support climate, risk-taking willingness, workplace belongingness

## Abstract

To remain competitive and viable in today’s rapidly evolving business landscape, organizations must strengthen their innovation capabilities. Innovative employee behavior often arises from disrupting established norms and deviating from conventional practices, making it crucial for organizations to focus on positive deviance. This study adopted organizational support theory to propose a parallel mediation model demonstrating how organizational support climate influences employees’ positive deviance. Based on a questionnaire survey distributed to 459 employees from Beijing, China, this study found that risk-taking willingness and workplace belongingness mediated the relationship between the organizational support climate and employees’ positive deviance. This study provides novel perspectives on the mechanisms of such behavior by highlighting that risk-taking willingness is a crucial factor in fostering positive deviance and that workplace belongingness plays an important role in bridging the two.

## 1. Introduction

To remain competitive and viable in today’s rapidly evolving business landscape, organizations must strengthen their innovation capabilities, which are closely linked to employees’ innovative abilities. Innovative employee behavior is often initiated by disrupting established norms and deviating from conventional practices. Therefore, organizations must focus on employees’ positive deviance. Contrary to the narrow conceptualization of workplace deviance as a negative set of behaviors, recent studies have increasingly emphasized its positive side: positive deviance. Previous research has indicated that such behaviors can significantly foster positive organizational change (Luthans et al., 2008; Robbins & Galperin, 2010), promote innovation (L. Li & Wang, 2021), and enhance organizational performance (Mertens et al., 2016). More recently, scholars have framed such behavior as an ethical decision rooted in moral courage (Popescu et al., 2025) and highlighted specific forms, such as customer-oriented positive deviance, which can significantly enhance service quality and customer gratitude (Gong et al., 2020). These findings underscore the increasing research interest in positive deviance.

Diverse perspectives have been considered to reveal the antecedents of positive deviance at work. Research on the antecedents of employees’ positive deviance has focused on individual or psychological facilitators, such as self-efficacy (Spreitzer & Sonenshein, 2004) and psychological empowerment (Appelbaum et al., 2007), as well as on varied leadership styles, such as benevolent leadership and moral leadership (L. Li & Wang, 2021). As an important situational factor, the organizational support climate has also been explored in previous studies regarding its relationship with employees’ positive deviance. For instance, Wayne et al. (1997) reported a positive correlation between organizational citizenship behaviors and perceived organizational support. Similarly, Kura et al. (2016) observed that perceived organizational support could increase positive deviance via organizational trust. In addition, Cohen and Ehrlich (2019) proposed a positive relationship between an organization’s supportive innovation climate and interpersonal positive deviance. Although previous empirical research has examined the influence of organizational support climate on the probability of positive deviance among employees, insufficient attention has been paid to the fundamental reason why organizational support climate fosters positive deviance in the workplace. The primary obstacle to positive deviance within a group context is the social risk linked to contravening established norms (Mertens et al., 2016; Warren, 2003). Psychological readiness to engage in positive deviance is reflected in the willingness to take social risks. Generally, employees may not feel sufficiently motivated to initiate change, especially through proactive, creative behaviors, due to the associated risks and uncertainties (N. Li et al., 2017).

This study considered these perspectives and explored the ways the organizational support climate affects employees’ positive deviance. Specifically, by examining the interaction between individual differences and the organizational environment, this study established a parallel mediation model grounded in organizational support theory. The model proposes that risk-taking willingness and workplace belongingness mediate the relationship between the organizational support climate and positive deviance. Drawing on an employee survey administered in the Beijing area, China, this study verified the mechanism by which organizational support climate indirectly promotes positive deviance by enhancing employees’ willingness to take risks and workplace belongingness.

This study, therefore, made a meaningful theoretical contribution to understanding the antecedents of positive deviance. By introducing organizational support climate as an independent variable and exploring its positive impact on positive deviance, this study revealed that risk-taking willingness and workplace belongingness partially mediate this relationship. In addition, this study offered new insight into how positive deviance can be enhanced. Through the parallel mediation model, this study indicated that risk-taking willingness is crucial in fostering positive deviance, while workplace belongingness plays an important role in bridging the two. Finally, this study expanded research on organizational support theory in the field of positive deviance and offered a new perspective for further development of the theory.

**Figure 1:**
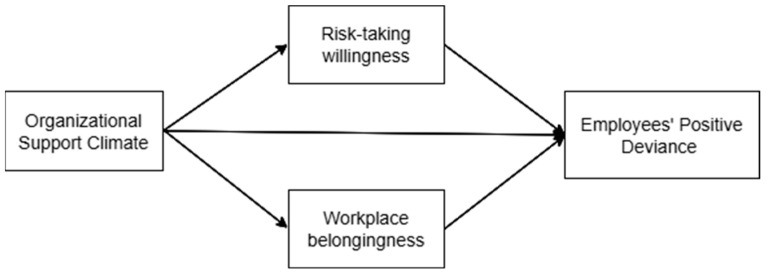
The study model.

Figure 1 illustrates the study model.

## 2. Theoretical Foundations and Hypothesis Formulation

### 2.1. Positive Deviance and Organizational Support Climate

Research on deviance originated from the French sociologist Émile Durkheim in the late 19th century (Longhofer & Winchester, 2016). While early research primarily investigated negative behaviors such as criminality and rule violations (Akers, 1968; Robinson & Bennett, 1995), the field has broadened to highlight constructive aspects, notably positive deviance. Positive deviance, also referred to as constructive deviance, refers to intentional behaviors that depart from organizational norms in honorable ways, aiming to produce beneficial outcomes for the organization (Galperin, 2012; Spreitzer & Sonenshein, 2004). In today’s rapidly evolving business landscape, organizations must strengthen their innovation capabilities. Since innovative behavior frequently arises from the disruption of established norms, focusing on employees’ positive deviance is critical for organizational viability. Recent systematic reviews indicate a surging scholarly interest in positive deviant behavior, positioning it as a critical emerging domain in organizational studies (Popescu et al., 2025).

To foster such constructive behaviors, the organizational context plays a pivotal role. Extensive research recognizes the organizational climate as a fundamental element that influences individual behavior by shaping specific outlooks toward the consequences of actions (Nyström, 1990; Park & Jo, 2018). Specifically, the organizational support climate refers to employees’ aggregate perceptions of the broad support they receive from the entire working environment, including supervisors, peers, and various departments (Lee et al., 2018; Luthans et al., 2008). Organizational support theory (OST) provides a framework for explaining how a supportive climate promotes positive deviance. Eisenberger et al. (1986) proposed that employees view the organization as a living entity with a purpose. Through this personification, employees develop beliefs regarding the extent to which the organization appreciates their contributions and prioritizes their well-being. When employees perceive a high level of support (i.e., a strong organizational support climate), it satisfies their socio-emotional needs, including approval, affiliation, and esteem (Eisenberger et al., 2020).

According to the reciprocity norm within OST, this perceived support creates a felt obligation to care for the organization’s welfare and help it achieve its objectives (Kurtessis et al., 2017). Consequently, employees are motivated to go beyond standard job responsibilities (in-role performance) and engage in extra-role behaviors that benefit the organization. Since positive deviance often involves voluntary acts that exceed standard expectations to solve problems or innovate, a supportive climate provides the necessary psychological safety and motivation for such behaviors. For instance, Haholongan and Kusdinar (2019) found that a participative climate positively influenced innovative behavior. When employees perceive the environment as supportive, they are more likely to take creative risks and deviate from conventional practices for the greater good, anticipating that their heightened efforts will be recognized and rewarded (Kurtessis et al., 2017). Based on this theoretical reasoning, we posit that a supportive climate fosters the necessary conditions for employees to engage in positive deviance. Thus, the following hypothesis is proposed:

**H1.**  *The organizational support climate has a positive relationship with employees’ positive deviance.*

### 2.2. Mediation of Risk-Taking Willingness

Risk-taking willingness refers to an individual’s propensity to engage in behaviors that involve uncertainty and potential negative consequences but also offer the possibility of positive outcomes (Sitkin & Pablo, 1992). In this study, we focus specifically on the willingness to take career-related risks to achieve organizational goals. An organizational support climate is a critical antecedent of employees’ willingness to take risks. According to organizational support theory, when employees perceive high levels of support (both instrumental and emotional), they develop a sense of psychological safety and trust in the organization (Eisenberger et al., 1986; Kurtessis et al., 2017). A supportive climate acts as a “safety net,” signaling to employees that well-intentioned failures will not be severely punished but rather viewed as learning opportunities (Edmondson, 1999). This perceived safety reduces the potential costs associated with risky behaviors. When employees feel their organization values their contributions and cares for their well-being, they are more likely to reciprocate by stepping out of their comfort zones and engaging in risky but potentially beneficial behaviors, rather than sticking to safe, routine procedures (Neves & Eisenberger, 2014).

Moreover, heightened risk-taking is a prerequisite for positive deviance. Positive deviance inherently involves violating established norms or rules to achieve honorable goals (Spreitzer & Sonenshein, 2004). This rule-breaking component introduces a unique layer of normative conflict and potential punitive consequences that standard creative tasks do not usually entail (Dahling et al., 2012). Popescu et al. (2025) highlighted that positive deviance demands the ‘moral courage’ to navigate the risks of defying established norms. According to the normative conflict model (Dahling et al., 2012), employees face a dilemma: either adhere to organizational norms or break them to solve problems. Engaging in such behavior carries significant social risks, such as rejection by peers or disciplinary action by supervisors (Mertens et al., 2016). Therefore, organizational support alone is not enough; it must be translated into an individual’s internal willingness to navigate these risks. Employees with a high willingness to take risks are less inhibited by the fear of sanctions and are more driven by the potential for positive change. They view the supportive climate not just as a comfort zone, but as a license to act. Thus, risk-taking willingness serves as the essential behavioral engine that converts the potential safety provided by the organization into the actual act of challenging the status quo. According to Kim and Choi (2018), risk-taking willingness acts as the motivational engine. It converts the potential derived from individual differentiation into actualized positive deviance. Without this specific propensity to tolerate the uncertainty of rule-breaking, even a supportive environment may not be sufficient to trigger positive deviance. Thus, the following hypothesis is proposed:

**H2.**  *Risk-taking willingness mediates the relationship between the organizational support climate and employees’ positive deviance.*

### 2.3. Mediation of Workplace Belongingness

The yearning for belonging is a fundamental human desire and one of the most potent motivators of individual effort (Maslow, 1954). Hagerty et al. (1992) described a sense of belongingness as being so deeply involved in a system or environment that one feels indispensable to it. Prior research has treated belongingness as a general construct, thereby overlooking its significance in specific social contexts, such as the workplace. Workplace belongingness provides a deeper understanding of identification and engagement in organizational life (Jena & Pradhan, 2018; Somoray et al., 2017). For example, workplace belongingness promotes employees’ feelings of acceptance and respect, which, in turn, enhances their proactivity and engagement in organizational activities (Jena & Pradhan, 2018). According to organizational support theory, perceived organizational support may fulfill employees’ socio-emotional needs, such as affiliation, thus leading to identification with the organization (Kurtessis et al., 2017). Employees who align with the organization may subsequently cultivate values akin to those of the organization (Kurtessis et al., 2017), leading them to reciprocate by exhibiting extra-role behavior at work. Cavanagh et al. (2021) showed that enhancing employees with intellectual disabilities’ feelings of belonging can lead to increased engagement and extra-role behaviors.

However, positive deviance requires more than just high engagement; it demands a willingness to challenge the status quo. Employees may lack sufficient drive to foster change, particularly proactively creative behaviors, due to the associated risks and uncertainties (N. Li et al., 2017). Cavanagh et al. (2021) emphasized that, despite its good intentions, positive deviance often imposes high psychological costs on employees, such as role stress and feelings of guilt, due to its inherent violation of organizational norms. As an important psychological state, this study proposes that workplace belongingness may serve as a bridging factor between the organizational support climate and positive deviance. Research by Dahling and Gutworth (2017) suggested that employees with a strong organizational identity act as “loyal rebels.” Possessing a profound sense of psychological ownership, employees prioritize the organization’s overall well-being over uncritical adherence to rules. When established norms hinder productivity or service quality, employees experience a form of constructive tension. Employees’ strong sense of belonging compels them to prioritize organizational goals over bureaucratic rules, emboldening them to engage in positive deviance, exemplified by breaking inefficient procedures to solve problems despite the inherent risks (Popescu et al., 2025). Thus, the following hypothesis is proposed:

**H3.**  *Workplace belongingness mediates the relationship between the organizational support climate and employees’ positive deviance.*

## 3. Methodology

### 3.1. Sample and Procedure

To investigate the impact of organizational support climate on employees’ positive deviance, we employed a quantitative analysis approach. A questionnaire was used to collect data from 459 employees in Beijing, China. The survey’s target population was randomly selected from a reliable online platform and included individuals from diverse industries. Before the survey was initiated, participants received instructions on the research purpose and procedures, as well as any specific research hypotheses that had not already been disclosed. Simultaneously, all participants were apprised of their ability to withdraw from the survey at any moment. The data did not contain any information that would identify an individual. The study process involved pretesting and formal testing. All questionnaire items were assessed for validity and comprehensibility by the pretest. Sixty pretest questionnaires were sent; 49 were collected, examined, and revised to form the final questionnaire. A total of 459 employees completed questionnaires, with a response rate of 86.44%.

Among the 459 respondents, 49.7% were male and 50.3% were female. The age distribution was as follows: 20–30 years (26.4%), 31–40 years (41.4%), 41–50 years (19.2%), and 51–60 years or more (13%). Moreover, 34% had only finished middle school or below. College graduates comprised 66% of the study population, while 11.8% had a master’s degree, and 3.5% had a doctorate. Regarding their tenure, 16.8% had worked for less than a year; 8.9% for 1 to 5 years; 22% for 6 to 10 years; 37.9% for 11 to 15 years; and 14.4% for more than 15 years. In terms of positions, 22.4% were directors and executives with supervisory responsibilities, and 77.6% were without such a post. Regarding employers, 46.2% were employed by private corporations, 39.4% by state-owned businesses, and 14.4% by the government or public institutions.

### 3.2. Measures

In accordance with accepted best practices for survey translation, the survey items were translated from English to Mandarin and back again (Brislin, 1970). Each item was anchored on a 5-point Likert scale ranging from 1 (strongly disagree) to 5 (strongly agree).

Positive Deviance: The nine-item scale developed by Galperin (2012) was used to measure positive deviance. Sample items include “Developed creative solutions to problems” and “Contravened my supervisor’s directives to enhance performance.” The Cronbach’s alpha for this scale was 0.90.

Organizational Support Climate: Thirteen items developed by Eisenberger et al. (1986) and Luthans et al. (2008) were used to measure the organizational support climate. Some examples included “When I have a problem, help is available from the organization” and “In this organization, the leadership acknowledges our capacity for innovative functioning.” The Cronbach’s alpha for this scale was 0.87.

Workplace Belongingness: Twelve items developed by Jena and Pradhan (2018) were used to measure workplace belongingness. Some examples included “My coworkers and I have many common themes in the work unit,” and “My organization strives to make my work as engaging and meaningful as possible.” The Cronbach’s alpha for this scale was 0.86.

Risk-taking Willingness: A four-item shortened version of the scale developed by Gomez-Mejia and Balkin (1989) was used. Sample items include “Not willing to take risks when selecting a position or an employer” and “Rather than a job with high risks and great rewards, I prefer a low-risk, high-security position that offers a consistent paycheck.” Items were reverse-coded so that higher scores indicate a greater willingness to take risks. The Cronbach’s alpha for this scale was 0.91.

Control Variables: The demographic variables listed below were incorporated as control variables in all analyses: gender, age, education, tenure, job title, and enterprise type.

Construct Validity and Reliability: To account for common method bias (CMB), we conducted Harman’s single-factor test, as all variables were derived from employee responses. The first factor accounted for 25.99% of the variance, which is below the 50% critical threshold (Podsakoff et al., 2003). Moreover, the confirmatory factor analysis showed that the single-factor model (CFI = 0.596; TLI = 0.568, RMSEA = 0.164) did not fit the data well. The results indicated that common-method variance was not a significant concern in this study and that the research findings were not affected.

To assess the empirical distinctiveness of the scales used in this study, we conducted CFA using Mplus 8.0. The CFA results are shown in Table 1. The normed Chi-square (χ^2^/df) was 3.756 (χ^2^ = 1419.7, df = 378), which is slightly higher than the prescribed cutoff of 3.00. However, some researchers have also accepted ratio values of 4.00 or 5.00 as indicative of a “good data model fit” (Mueller, 2012). The acquired CFI and TLI values were 0.915 and 0.903, respectively. The fit indices surpass the stipulated threshold of 0.90 (Bentler & Bonett, 1980), hence validating the indicators of model fit. A value below 0.08 signifies an acceptable degree of approximation error among the proposed variables (Browne & Cudeck, 1992). We obtained an RMSEA of 0.068, indicating a good fit. Therefore, based on these guidelines, the acquired value for this study signifies a satisfactory match. The CFA indicators met the verification standards used to assess the appropriateness of the postulated measurement model for the data. In addition, the four-factor model’s fit was assessed against three other models, demonstrating its superiority.

Convergent and discriminant validity were evaluated using average variance extracted (AVE) and composite reliability (CR). As shown in Table 1, all CR values exceeded 0.70, and all AVE values were above 0.50, supporting convergent validity (Fornell & Larcker, 1981). Discriminant validity was also established, as the square root of the AVE for each construct (diagonal elements in Table 2) was greater than its correlation with any other construct.

## 4. Result

### 4.1. Correlation Analysis

A correlation analysis was performed using SPSS 18.0. Descriptive statistics for all research variables are presented in Table 2. Preliminary evidence for all the predicted correlations was provided by the significant correlations among the important variables.

### 4.2. Tests of Hypotheses

In this study, SPSS version 18.0 was used to evaluate the proposed hypotheses. H1 posits that the organizational support climate is positively related to employees’ positive deviance. As demonstrated by the regression analysis, the total effect (c) was significant (β = 0.644, *p* < 0.001); thus, Hypothesis 1 was supported.

**Figure 2:**
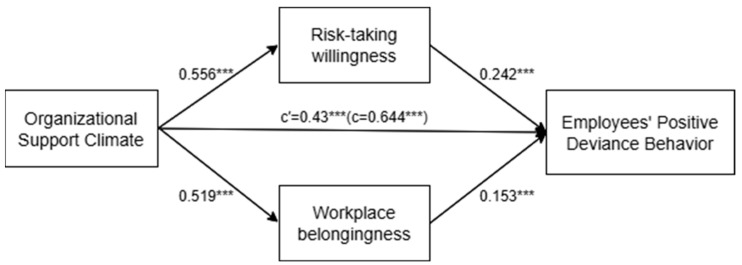
The variables path coefficient diagram. Note: Standardized path coefficients are reported. *c* represents the total effect, and $c^{\prime }$ represents the direct effect after controlling for the mediator. *** *p* < 0.001.

H2 and H3 suggest that risk-taking willingness and workplace belongingness mediate the relationship between organizational support climate and employees’ positive deviance. Hypotheses 2 and 3 were tested using a bootstrapping-based mediation test in PROCESS Model 4 (Hayes, 2017). The bootstrapped indirect impact is deemed significant if the bias-corrected 95% confidence interval (CI) does not encompass zero. The path coefficient diagram between variables is shown in Figure 2.

As shown in Table 3, when the variable was WTR, the results that do not depend on the normal sampling distribution assumption indicate a coefficient of 0.107, with a standard error of 0.021. The 95% confidence interval (CI) based on 5000 bootstrapped samples did not include zero (lower limit: 0.066; upper limit: 0.151). Thus, Hypothesis 2 was supported.

As shown in Table 3, when the variable was WB, the results that do not depend on the normal sampling distribution assumption indicate a coefficient of 0.063, with a standard error of 0.019. The 95% confidence interval (CI) based on 5000 bootstrapped samples did not encompass zero (low limit 0.025, upper limit 0.10). Thus, Hypothesis 3 was supported.

Despite the significant indirect effects via risk-taking willingness and workplace belongingness, the direct effect of organizational support climate on employees’ positive deviance remained statistically significant (B = 0.34, *p* < 0.001). These results supported a partial mediation model. To quantify the mediation effect, we calculated the variance accounted for (VAF). The combined indirect effects account for approximately 33.2% of the total effect. Specifically, risk-taking willingness (effect = 0.107, 95% CI [0.066, 0.151]) mediates a larger portion of the effect compared to workplace belongingness (effect = 0.063, 95% CI [0.025, 0.100]).

## 5. Discussion

This study developed and tested a parallel mediation model grounded in organizational support theory (OST) to examine how the organizational support climate fosters positive deviance. The results confirmed that both risk-taking willingness and workplace belongingness serve as partial mediators in this relationship. This finding suggests that a supportive climate promotes positive deviance through the dual psychological pathways of risk-taking as a cognitive evaluation of safety, and belongingness as an emotional bond with the organization.

First, our findings reveal that risk-taking willingness mediates the link between organizational support climate and positive deviance. Positive deviance inherently involves violating established norms, which carries significant social risks and uncertainties (N. Li et al., 2017). A supportive climate acts as a “safety net,” signaling that well-intentioned failures will be tolerated rather than punished (Edmondson, 1999). This perceived psychological safety emboldens employees to step out of their comfort zones. Specifically, when employees perceive high organizational support, they are more willing to bear the potential costs of rule-breaking to achieve better outcomes, viewing the support not just as a comfort but as a license to act. In this context, organizational support mitigates the perceived risks of deviation, leading employees to view constructive deviation as a rational and beneficial decision.

Second, workplace belongingness plays a crucial bridging role. While risk-taking addresses the capacity to deviate, belongingness addresses the motivation to do so. As noted in our results, positive deviance requires more than just high engagement; it demands a willingness to challenge the status quo. Driven by a profound sense of psychological ownership, employees do not uncritically adhere to rules; rather, they are motivated by the organization’s overall well-being. Their strong sense of belonging compels them to prioritize organizational goals over bureaucratic rules, emboldening them to engage in positive deviance, exemplified by breaking inefficient procedures to solve problems despite the inherent risks (Popescu et al., 2025). This suggests that employees with high belongingness act as “loyal rebels” (Dahling & Gutworth, 2017), breaking rules not out of defiance, but out of deep care for the collective good.

In summary, these two mediators function in complementary ways. Willingness to take risks reduces the fear of consequences, while workplace belongingness enhances the desire to contribute. The parallel mediation suggests that to effectively foster positive deviance, organizations must simultaneously cultivate a safe environment and a cohesive community.

Furthermore, the results indicated a partial mediation effect. As noted by Hayes (2017), the persistence of a significant direct effect after the inclusion of mediators should be treated not merely as a statistical outcome but as evidence of theoretical incompleteness. This suggests that while the proposed mediators serve as crucial mechanisms, they are not the sole pathways linking organizational support climate to positive deviance, and additional unmodeled mechanisms likely exist. For instance, according to self-determination theory (Ryan & Deci, 2002), a supportive climate fulfills employees’ basic need for autonomy. When employees feel their autonomy is supported, they are more likely to internalize the organization’s goals and use their judgment to solve problems (Caesens & Stinglhamber, 2014). From a role theory perspective, high organizational support may enhance employees’ self-efficacy regarding their role breadth (Parker, 1998). Supported employees tend to define their roles more broadly, seeing themselves not just as rule-followers but as proactive problem solvers. This enhanced sense of efficacy directly empowers them to handle the complexity of rule-breaking behavior. This highlights that organizational support acts as a comprehensive resource that both indirectly facilitates and directly empowers extra-role behaviors.

### 5.1. Theoretical Implications

This study makes several contributions to the existing literature on organizational behavior and positive deviance. First, we advanced the theoretical boundaries of organizational support theory by applying it to the domain of positive deviance. While prior research has predominantly focused on individual traits or specific leadership styles as antecedents of positive deviance (Appelbaum et al., 2007; L. Li & Wang, 2021; Spreitzer & Sonenshein, 2004), our study offered a fresh perspective by highlighting the pivotal role of the broader organizational context. This climate-oriented perspective advanced existing behavioral research. It demonstrated that a supportive environment is fundamental for empowering employees to challenge norms for the collective good. By integrating the concept of organizational support climate with positive deviance, we unveiled previously unseen insights into how an organization can systematically cultivate the moral courage required for positive rule-breaking (Eisenberger et al., 2020; Popescu et al., 2025).

Second, our findings revealed the dual psychological mechanisms of risk-taking willingness and workplace belongingness, which inherently affected employees’ decisions to deviate positively. This insight extended the reciprocity norm proposed in organizational support theory, which traditionally emphasized general obligation (Eisenberger et al., 1986). We distinguished between the cognitive mechanism of risk-taking willingness, which reduced the fear of sanctions, and the emotional mechanism of workplace belongingness, which fueled the desire to contribute. This added depth to our understanding of the “loyal rebel” phenomenon, where employees prioritize organizational welfare over bureaucratic adherence (Dahling & Gutworth, 2017). It suggested that positive deviance requires a unique psychological state in which the perceived safety to act and the affective commitment to the organization function in complementary ways.

Third, we enriched the understanding of organizational dynamics by demonstrating the complementary mediating roles of risk-taking willingness and workplace belongingness. Notably, the continued presence of a significant direct relationship indicates that organizational support acts as a comprehensive resource that transcends these psychological mechanisms. Specifically, beyond providing emotional assurance, our study suggested that organizational support drives a fundamental cognitive shift in role definition. Under this influence, employees expand their role boundaries, internalizing problem-solving as a core duty rather than an optional extra-role behavior (Parker, 1998). This implied that a supportive climate not only offers safety but also enables autonomy and self-efficacy to translate directly into action. Therefore, it fostered a proactive workforce capable of navigating the complexities of innovation and norm-violation (Caesens & Stinglhamber, 2014).

### 5.2. Practical Implications

This study also offers valuable practical implications for organizational management. First, managers should prioritize cultivating a robust organizational support climate. Perceived support increases employees’ commitment to organizational goals (Eisenberger et al., 2020). Leaders must go beyond simple resource provision. They should actively build a psychological safety buffer (Edmondson, 1999). This approach ensures employees feel secure enough to deviate from established norms for the benefit of the organization. By integrating tangible support with emotional backing, managers can effectively empower positive deviance.

Second, management should actively foster a willingness to take risks by redefining the organizational response to failure. An error management culture encourages positive experimentation (Frese & Keith, 2015); leaders must demonstrate that well-intentioned mistakes are viewed as learning opportunities rather than punishable offenses. This perspective empowers employees to assess the safety of challenging the status quo (Anisman Razin et al., 2021; Sitkin & Pablo, 1992). Therefore, employees can take the necessary initiative to solve problems without the fear of negative repercussions.

Finally, organizations should leverage workplace belongingness as a motivational driver for positive change. Leaders are advised to adopt an inclusive leadership style to validate employee contributions (Carmeli et al., 2010; Roberson & Perry, 2022). Employees who challenge bureaucratic rules often possess a profound sense of psychological ownership (Dawkins et al., 2017; J. L. Pierce et al., 2001). Managers should recognize these individuals as loyal rebels acting for the collective good (Dahling & Gutworth, 2017). This recognition channels their deep organizational attachment into productive innovation and problem-solving.

### 5.3. Limitations and Future Scope

Although this study sheds light on the mechanism linking organizational support climate to employees’ positive deviance, its methodological limitations point to meaningful directions for future research. A primary limitation of this study is its geographically restricted sample, drawn predominantly from Beijing, which may limit the generalizability of the findings. To improve the generalizability of the findings, future studies are recommended to expand their sampling to include employees from diverse regions and industries. Specifically, Beijing’s unique cultural and economic environment may differ from that of other regions. This could influence the applicability of our results to different cultural contexts. Second, this study used cross-sectional data, which has limitations related to the time frame. Hence, this study should be replicated using longitudinal research to enable causal inference. We also wish to draw attention to the domain-specific nature of the risk-taking willingness measure. We utilized the scale adapted from Gomez-Mejia and Balkin (1989), which primarily captures risk preferences related to employment choices and job security (e.g., preferring a secure position over a high-risk, high-reward job). Therefore, this measure reflects a domain-specific inclination centered on career and financial stability, rather than a general, cross-domain personality trait or risk-taking behaviors in daily task execution. Although this domain-specific measure aligns with our focus on career-related risks associated with positive deviance, future research would benefit from incorporating general trait-based scales to capture a wider spectrum of risk-taking propensities.

Given the partial mediation findings, future studies should employ longitudinal or experimental designs to more rigorously disentangle the causal chains. Longitudinal data would allow researchers to observe how organizational support initially fosters psychological safety and belongingness over time, rather than triggering immediate behavioral changes, providing a more dynamic picture of the positive deviance process. In addition, as noted earlier, the partial mediation results indicate that the organizational support climate may influence positive deviance through other unmodeled mechanisms beyond risk-taking willingness and workplace belongingness. Future research should therefore extend our model by exploring additional mediators. For example, researchers could investigate affective mechanisms such as psychological empowerment or positive effects, which might also serve as bridges between support climate and deviant behaviors. Furthermore, exploring individual differences as potential moderators could help clarify when and for whom these mediation effects are strongest, thereby addressing the theoretical incompleteness highlighted by the partial mediation findings. Moreover, future research should explore the potential non-linear relationship between organizational support climate and positive deviance. While this study established a positive linear association, the Too-Much-of-a-Good-Thing effect described by J. R. Pierce and Aguinis (2013) suggests that beneficial antecedents may cause harm when they reach excessive levels. Harris and Kacmar (2018) identified an inverted U-shaped relationship between perceived organizational support and extra-role performance. An overwhelming level of support could inadvertently create a comfort zone that fosters complacency or generates an excessive sense of indebtedness (N. Li et al., 2017). In such an environment, employees might become reluctant to challenge the status quo or violate norms for fear of disrupting the harmonious relationship with the organization. Therefore, future studies should examine whether a tipping point exists where the benefits of a supportive climate diminish or reverse, thereby providing a more nuanced understanding of the boundary conditions of organizational support. Finally, while this study focused exclusively on positive deviance, future research could benefit from examining the dynamic relationship between deviance and positive deviance. Specifically, scholars could investigate the tipping point where positive rule-breaking might slide into deviance and identify the boundary conditions that distinguish these two forms of behavior.

## 6. Conclusions

This study establishes a dual-process model linking organizational support climate to positive deviance, offering a nuanced explanation of how organizations can cultivate “loyal rebels.” By integrating the complementary mechanisms of risk-taking willingness and workplace belongingness, our findings highlight that fostering constructive deviance requires a delicate balance: organizations must simultaneously provide a psychological safety net that encourages experimentation and a communal bond that ensures such behaviors are motivated by organizational interest.

Theoretically, this study contributes to the literature by moving beyond direct-effect models to reveal the complex cognitive and affective pathways underlying positive deviance. Furthermore, the identification of a partial mediation effect serves as a critical theoretical contribution in itself. It suggests that while risk-taking and belongingness are pivotal, the phenomenon of positive deviance is multifaceted. Our study, thus, provides a foundational framework that not only explains current mechanisms but also invites future research to explore additional unmodeled pathways, such as individual traits or distinct motivational states. Ultimately, this study advocates for a holistic approach to management—one that empowers employees to break rules constructively, not out of defiance, but out of a deep sense of safety and belonging.

## Figures and Tables

**Table 1:** Confirmatory factor analysis results.

Model	χ^2^	df	χ^2^/df	∆χ^2^ (∆df) ^4^	CFI	TLI	RMSEA
Research model (4 factor)	1419.7	378	3.756 ***		0.915	0.903	0.068
Alternative model 1 (3 factor) ^1^	4371.709	402	10.875 ***	2952.009 ***	0.67	0.643	0.106
Alternative model 2 (2 factor) ^2^	4867.034	404	12.047 ***	495.325 ***	0.63	0.601	0.111
Alternative model 3 (1 factor) ^3^	5390.176	406	13.276 ***	523.142 ***	0.596	0.568	0.164

Note. n = 459, *** *p* < 0.001, OSC = organizational support climate, WTR = willingness to take risks, WB = workplace belongingness, PDB = positive deviant behavior; ^1^ 3 factor: OSC+ WTR, WB, and PDB, ^2^ 2 factor: OSC+WTR+WB, and PDB, ^3^ 1 factor: OSC + WTR + WB + PDB, ^4^ Each model’s chi-square difference quantifies its deviation relative to the four-factor model.

**Table 2:** Descriptive statistics.

Variable	Mean	S. D	1	2	3	4	5	6	7 (OSC)	8 (WB)	9 (WTR)	10 (PDB)
1. Gender	1.50	0.50										
2. Age	2.19	0.97	−0.02									
3. Education	2.13	1.11	0.09 *	−0.15 **								
4. Job Tenure	3.27	1.33	0.05	0.70 **	0.06							
5. Job Title	1.37	0.79	−0.03	0.08	0.10 *	0.19 **						
6. Enterprise Type	2.21	0.99	0.10 *	0.05	0.29 **	0.13 **	0.08					
7. OSC	3.44	0.86	−0.04	0.32 **	0.03	0.37 **	0.11 *	0.02	***0.82***			
8. WB	3.51	0.75	0.01	0.29 **	0.01	0.40 **	0.08	0.03	0.59 **	***0.82***		
9. WTR	2.56	0.88	−0.13 **	0.27 **	−0.12	0.23 **	0.07	0.09 *	0.57 **	0.40 **	***0.80***	
10. PDB	3.34	0.68	−0.01	0.18 **	−0.03	0.28 **	0.09	−0.03	0.64 **	0.50 **	0.53 **	***0.78***
Average Variance Extracted									0.67	0.67	0.65	0.61
Composite Reliability									0.93	0.92	0.88	0.83

Note. N = 459; * *p* < 0.05 and ** *p* < 0.01; OSC = organizational support climate; WB = workplace belongingness; WTR = willingness to take risks; PDB = positive deviant behavior; the square root of the average variance extracted (AVE) is shown on the diagonal in bold italics.

**Table 3:** Analysis of the mediation effect of the bootstrap.

	Variable	Effect Value	Boot SE	LLCI	ULCI
Total effect		0.509	0.03	0.45	0.569
Direct effect		0.34	0.038	0.265	0.415
Indirect effect	WTR	0.107	0.021	0.066	0.151
	WB	0.063	0.019	0.025	0.10

Note. n = 459; WTR = risk-taking willingness; WB = workplace belongingness.

## Data Availability

Data will be available upon reasonable request exclusively for academic reasons from the authors.
